# Successful use of differential target multiplexed spinal cord stimulation for chronic postsurgical abdominal pain

**DOI:** 10.1097/PR9.0000000000001059

**Published:** 2023-01-19

**Authors:** Ryusuke Tanaka, Kenji Shinohara, Yohei Hidai, Chiaki Kiuchi, Satoshi Tanaka, Mikito Kawamata, Junichi Sasao

**Affiliations:** a Division of Anesthesiology, Ina Central Hospital, Ina City, Nagano, Japan; b Department of Anesthesiology and Resuscitology, Shinshu University School of Medicine; c Division of Orthopedic Surgery, Ina Central Hospital, Ina City, Nagano, Japan

**Keywords:** Chronic postsurgical pain, Spinal cord stimulation, Differential target multiplexed stimulation, Neuropathic pain

## Abstract

**Introduction:**

Recent advances in stimulation techniques have improved the efficacy and expanded the applicability of spinal cord stimulation (SCS). Among these techniques, there are no reports on the efficacy of differential target multiplexed (DTM) SCS for chronic postsurgical pain (CPSP) after abdominal surgery. Therefore, we present the successful use of DTM SCS for CPSP after distal pancreatectomy.

**Methods:**

A 49-year-old man with hypertension and severe chronic low back pain presented with neuropathic CPSP involving the left abdomen in the area of a laparotomy incision. His pain was refractory to conservative treatment and was rated 10 on a numerical rating scale (NRS). He underwent permanent implantation of a pulse generator after a 14-day trial stimulation.

**Results:**

Chronic postsurgical pain was well controlled (NRS 1–2) at a 3-month follow-up with DTM SCS.

**Conclusion:**

Differential target multiplexed SCS can be a new treatment option for neuropathic CPSP that is resistant to conservative treatment. It is important to further examine the characteristics of CPSP and identify appropriate candidates for the successful use of DTM SCS.

## Abstract 2

We report the successful use of differential target multiplexed spinal cord stimulation in a patient with refractory chronic postsurgical pain and neuropathic features after distal pancreatectomy.

## 1. Introduction

Chronic postsurgical pain (CPSP) is estimated to occur in 10% to 50% of surgical patients^14^; of whom, 2% to 10% experience severe CPSP, which results in functional impairment.^7,14^ Although various approaches to prevent and treat CPSP have been proposed,^3,5^ definitive treatment for CPSP has not been established.

Spinal cord stimulation (SCS) is a safe and effective treatment, particularly for mixed neuropathic/nociceptive and neuropathic/radicular pain conditions, such as failed back surgery syndrome and complex regional pain syndrome.^21^ However, recent advances in stimulation techniques, which have a mechanism of action distinct from traditional SCS, have improved the efficacy and extended the applicability of SCS.^21^

Differential target multiplexed (DTM) SCS is a new paresthesia-free stimulation technique that mediates its analgesic effect by means of modulating neuron–glial interaction.^20^ Although DTM SCS was shown to be superior to traditional tonic SCS in relieving low back pain (LBP),^6^ its efficacy for CPSP has not been reported. We report a case of neuropathic CPSP after distal pancreatectomy (DP) with severe chronic LBP resistant to conservative treatment. The broad trunk pain was challenging to treat with conventional tonic SCS.^10^ Therefore, we used DTM SCS, expecting concomitant remission of the CPSP and LBP. This is a case report of the successful use of DTM SCS for refractory CPSP after abdominal surgery. Written informed consent for publication was obtained from the patient.

## 2. Methods

A 49-year-old man (59 kg, 157 cm) with hypertension and severe chronic LBP presented with persistent pain involving the left abdomen around a transverse incision. He had undergone DP for a pancreatic pseudocyst 7 years earlier. Surgery was performed under general anesthesia and epidural anesthesia. He had left abdominal pain after surgery, which did not recur at the subsequent outpatient follow-up. He was referred to the pain clinic 1 year after the surgery. His pain was characterized by numbness, tingling, and burning radiating over the T8-9 dermatomes, including the surgical wound. He also had hypoesthesia in the region with pain. He experienced electric shock–like pain attacks with constant pain. His pain scored 21 on the painDETECT questionnaire-Japanese version (PDQ-J),^16^ indicating that his pain had a neuropathic etiology. Computed tomography of the abdomen and magnetic resonance imaging (MRI) of the thoracic and lumbar spinal cord did not reveal the cause of his pain. Over several years, his pain was treated with medications (duloxetine 20 mg/d, mirogabalin 30 mg/d). However, his pain worsened, and he experienced constant pain of a rating of 6 to 8 on a numerical rating scale (NRS) with pain attack (NRS 10), requiring tramadol up to 300 mg/d. A thoracic epidural block was performed, although the effect was temporary. We also performed a transversus abdominis plane block, which alleviated his pain partially. However, severe pain persisted in his left abdomen, and the conservative treatment was ineffective. The temporary remission of his pain by the epidural block had implied that his neuropathic CPSP was derived from a peripheral component. Therefore, we offered SCS treatment because it is an effective treatment for peripheral neuropathic pain.^17^

**Figure 1.**
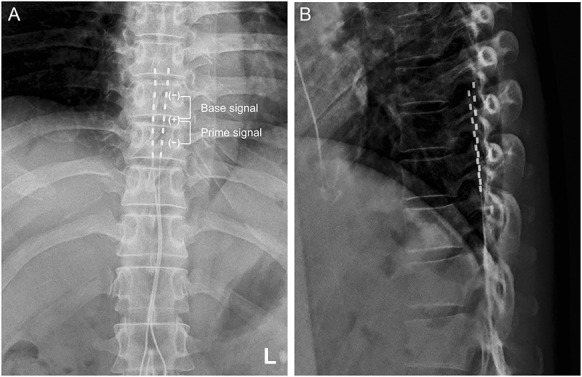
Anteroposterior (A) and lateral (B) view of two 8-contact leads. The tips of the leads were placed at the bottom of the T7 vertebral body. This image represents the final placement of the leads. Differential target multiplexed stimulation consisted of a base signal (frequency, 50 Hz; pulse width 200 μs) and other 3 programs of prime signals (frequency, 300 Hz; pulse width 170 μs).

The trial leads were inserted in the operating room. The patient was placed in the prone position. A 14-gauge Tuohy needle was inserted at the L1-2 left paramedian interspace. We confirmed that the needle tip was in the epidural space using fluoroscopy and loss of resistance. A single 8-contact lead (Vectris SureScanMRI, Medtronic Inc, Minneapolis, MN) was advanced into the epidural space. Tonic stimulation was then performed. The tip of lead was placed at the bottom of the T7 vertebral body, confirming paresthesia by tonic stimulation over the region of pain. Another lead was inserted through the L1-2 right paramedian interspace in the same way, and the tip of the lead was placed at the bottom of T7. During the 14-day trial, we tested tonic stimulation (frequency, 50 Hz; pulse width, 200 μs; pulse intensity, 3.4 mA), and DTM stimulation, which consisted of a base signal (frequency, 50 Hz; pulse width 200 μs) and other 3 programs of prime signals (frequency, 300 Hz; pulse width 170 μs). The intensity of DTM was set at 70% and 65% of the threshold level of paresthesia for a base signal (1.4 mA) and a prime signal (1.2 mA), respectively, according to the manufacturer's instructions. We applied DTM stimulation for 7 days, followed by tonic stimulation for the next 7 days. Immediately after applying DTM stimulation, the patient's left abdominal pain was alleviated (NRS 0), and the effectiveness of SCS was maintained with tonic stimulation. Therefore, the patient was offered permanent implantation of a pulse generator. In the operating room, the trial leads were removed, and permanent implantation of the leads was performed in the same manner: two 8-contact leads were inserted at the T12-L1 level, and the tips of the leads were placed at the bottom of the T7 vertebral body (Fig. 1A and B). A pulse generator (Intellis; Medtronic Inc, Minneapolis, MN) was then inserted into the left buttocks.

## 3. Results

After the permanent implantation of a pulse generator, DTM stimulation was applied to the SCS. The intensity of DTM stimulation was decided in a similar manner as trial stimulation and set at 2.3 mA and 2.6 mA for the base and prime signals, respectively. Although the patient's hypoesthesia in the region of pain did not remit, the left abdominal pain was completely alleviated (NRS 0) with tramadol 100 mg/day and duloxetine 20 mg/d, achieving a concomitant remission of LBP. At the 3-month follow-up, the left abdominal pain remained relived with occasional pain attacks (NRS 1–2), which remitted spontaneously and rarely troubled him. However, his LBP was slightly worse and tramadol 150 mg/d and duloxetine 20 mg/d were administered.

## 4. Discussion

In this case, we confirmed that DTM SCS alleviated CPSP after abdominal surgery. Studies on SCS for CPSP are very limited; thus, the modalities of SCS that may be suitable for the treatment of CPSP have not been established yet. Recently, paresthesia-free SCS, including DTM SCS, was shown to provide more effective pain relief compared with tonic SCS in other pain conditions.^6,11^ Among these SCS techniques, 10-kHz SCS was reported to decrease the mean pain score by >70% in more than 70% of patients with CPSP.^2,9,12^ In our case, DTM SCS achieved 80% to 90% pain reduction of the CPSP on NRS at 3 months, which implies that DTM SCS is comparable to10-kHz SCS for the treatment of CPSP. Therefore, DTM SCS, similar to 10-kHz SCS, is expected to be a more optimal treatment option than tonic SCS, considering its efficacy, which is approximately a 50% pain reduction in the pain score of 50% of patients with other pain conditions.^19^ However, it should be noted that it was unclear in our case whether tonic SCS or DTM SCS was superior for treatment. In addition, we must confirm the effectiveness of DTM SCS over the years of follow-up because it has been reported that 12% of patients developed tolerance to tonic SCS over 10 years.^13^ The efficacy of DTM SCS on CPSP should be compared with those of other modalities of SCS in long-term follow-up studies.

The mechanism of action of DTM SCS is distinct from that of the traditional tonic SCS. Tonic SCS stimulates Aβ fibers in the dorsal column antidromically and orthodromically, thereby activating inhibitory interneurons and the descending pain modulation system, respectively.^18^ By contrast, DTM SCS modulates dorsal horn glial gene experession,^20^ which takes several days. However, we observed immediate pain relief by DTM SCS. A previous report showed that paresthesia-free SCS also inhibited somatosensory evoked potentials immediately like tonic SCS.^1^ This fact suggests that paresthesia-free SCS also activates Aβ fibers in the dorsal column, leading to blockage of impulses from the periphery.^1^ We speculate that DTM SCS mediates its analgesic effect through not only modulating neuron–glial interaction but also activating inhibitory neural pathways, leading to immediate pain relief. Further studies are needed to clarify the mechanism of action of DTM SCS.

Chronic postsurgical pain is derived from several distinct mechanisms (nociceptive, inflammatory, and neuropathic).^5^ Among these, neuropathic pain is related to more severe and persistent pain, which results in a poorer quality of life.^7,15^ To evaluate the neuropathic features of pain, we used the PDQ-J, which is a Japanese version of the PDQ.^16^ A PDQ score of ≥19 indicates that a neuropathic component is likely (>90%).^8^ In this case, the PDQ-J score was 21, which implied that a neuropathic component contributed to forming CPSP. At this point, good indications for DTM SCS are not clarified, although peripheral neuropathic pain can be a good responder to DTM SCS considering its mode of action.^4,20^ In our case, pathological features may contribute to the successful treatment of CPSP. However, paresthesia-free SCS was reported to effectively relieve nonneuropathic abdominal pain.^12^ Thus, the effectiveness of DTM-SCS on nonneuropathic CPSP should be examined, and the most appropriate candidate for DTM-SCS should be determined.

In conclusion, refractory CPSP with neuropathic characteristics was alleviated by DTM SCS treatment. Further studies are needed to clarify good candidates for DTM SCS and to confirm an optimal stimulation technique for the treatment of CPSP.

## Disclosures

The authors have no conflicts of interest to declare.
